# Supplementary material for: Scattering approach to diffusion quantifies axonal damage in brain injury
Source: Nat Commun. 2025 Nov 6;16:9808. doi: 10.1038/s41467-025-64793-1 (PMC12592534; doi:10.1038/s41467-025-64793-1)
Supplement: Supplementary file 2 — Reporting Summary [file 41467_2025_64793_MOESM2_ESM.pdf]

nature portfolio

Corresponding author(s): Ali Abdollahzadeh

Last updated by author(s): Sep 15, 2025

Reporting Summary

Nature Portfolio wishes to improve the reproducibility of the work that we publish. This form provides structure for consistency and transparency in reporting. For further information on Nature Portfolio policies, see our Editorial Policies and the Editorial Policy Checklist.

Statistics

For all statistical analyses, confirm that the following items are present in the figure legend, table legend, main text, or Methods section.

n/a	Confirmed	
☐	☒	The exact sample size (*n*) for each experimental group/condition, given as a discrete number and unit of measurement
☐	☒	A statement on whether measurements were taken from distinct samples or whether the same sample was measured repeatedly
☐	☒	The statistical test(s) used AND whether they are one- or two-sided<br>*Only common tests should be described solely by name; describe more complex techniques in the Methods section.*
☒	☐	A description of all covariates tested
☒	☐	A description of any assumptions or corrections, such as tests of normality and adjustment for multiple comparisons
☐	☒	A full description of the statistical parameters including central tendency (e.g. means) or other basic estimates (e.g. regression coefficient) AND variation (e.g. standard deviation) or associated estimates of uncertainty (e.g. confidence intervals)
☐	☒	For null hypothesis testing, the test statistic (e.g. *F*, *t*, *r*) with confidence intervals, effect sizes, degrees of freedom and *P* value noted<br>*Give P values as exact values whenever suitable.*
☒	☐	For Bayesian analysis, information on the choice of priors and Markov chain Monte Carlo settings
☒	☐	For hierarchical and complex designs, identification of the appropriate level for tests and full reporting of outcomes
☐	☒	Estimates of effect sizes (e.g. Cohen's *d*, Pearson's *r*), indicating how they were calculated

Our web collection on statistics for biologists contains articles on many of the points above.

Software and code

Policy information about availability of computer code

Data collection	Segmentation of white matter microstructure in 3d electron microscopy datasets is publicly available at https://etsin.fairdata.fi/dataset/f8ccc23a-1f1a-4c98-86b7-b63652a809c3.<br>Axonal morphology and time-dependent diffusion MRI data in brain injury that support the findings of this study are publicly available at https://etsin.fairdata.fi/dataset/7ab3737d-0884-400e-ab57-657e3667d52b.<br>Source data are provided with this paper.
Data analysis	The source code of DeepACSON software is publicly available at https://github.com/aAbdz/DeepACSON.<br>The source code of the Monte Carlo simulator (the RMS package) is publicly available at https://github.com/NYU-DiffusionMRI.<br>The source code used to generate the results presented in the manuscript is publicly available at https://github.com/aAbdz/scattering-to-diffusion.

For manuscripts utilizing custom algorithms or software that are central to the research but not yet described in published literature, software must be made available to editors and reviewers. We strongly encourage code deposition in a community repository (e.g. GitHub). See the Nature Portfolio guidelines for submitting code & software for further information.

Data

Policy information about availability of data

All manuscripts must include a data availability statement. This statement should provide the following information, where applicable:

- Accession codes, unique identifiers, or web links for publicly available datasets
- A description of any restrictions on data availability
- For clinical datasets or third party data, please ensure that the statement adheres to our policy

All datasets and software that support the findings of this study are either publicly. These statements are provided in the manuscript: Data and Code Availability statements.

Research involving human participants, their data, or biological material

Policy information about studies with human participants or human data. See also policy information about sex, gender (identity/presentation), and sexual orientation and race, ethnicity and racism.

Reporting on sex and gender	NA
Reporting on race, ethnicity, or other socially relevant groupings	NA
Population characteristics	NA
Recruitment	NA
Ethics oversight	NA

Note that full information on the approval of the study protocol must also be provided in the manuscript.

Field-specific reporting

Please select the one below that is the best fit for your research. If you are not sure, read the appropriate sections before making your selection.

☒ Life sciences ☐ Behavioural & social sciences ☐ Ecological, evolutionary & environmental sciences

For a reference copy of the document with all sections, see nature.com/documents/nr-reporting-summary-flat.pdf

Life sciences study design

All studies must disclose on these points even when the disclosure is negative.

Sample size	We imaged the white matter (corpus callosum and cingulum) of five rat brains, two sham-operated rats and three rats with traumatic brain injury (TBI), using 3D electron microscopy (3D-EM). The images were acquired ipsi- and contra-lateral to the site of injury, resulting in a total of 20 EM datasets. Each 3D-EM dataset included thousands of myelinated axons that we evaluated individually to draw our statistical conclusions.<br>Given the substantial investments necessary to acquire 3D-EM datasets and their analysis, this sample represents one of the largest, if not the largest, 3D-EM white matter datasets reported to date.<br>We also conducted an ex vivo time-dependent diffusion MRI experiment on two sham-operated rats and three rats with mild TBI, sacrificed four weeks post-surgery, to experimentally validate our theoretical predictions.
Data exclusions	No data were excluded.
Replication	NA
Randomization	NA
Blinding	NA

Reporting for specific materials, systems and methods

We require information from authors about some types of materials, experimental systems and methods used in many studies. Here, indicate whether each material, system or method listed is relevant to your study. If you are not sure if a list item applies to your research, read the appropriate section before selecting a response.

Materials & experimental systems

n/a	Involved in the study
☒	☐ Antibodies
☒	☐ Eukaryotic cell lines
☒	☐ Palaeontology and archaeology
☐	☒ Animals and other organisms
☒	☐ Clinical data
☒	☐ Dual use research of concern
☒	☐ Plants

Methods

n/a	Involved in the study
☒	☐ ChIP-seq
☒	☐ Flow cytometry
☐	☒ MRI-based neuroimaging

Animals and other research organisms

Policy information about studies involving animals; ARRIVE guidelines recommended for reporting animal research, and Sex and Gender in Research

Laboratory animals: Adult male Sprague-Dawley rats, 8-10 weeks old.

Wild animals: NA

Reporting on sex: To reduce within and between groups structural variabilities in white matter tissues, the study only includes male rats.

Field-collected samples: NA

Ethics oversight: All animal procedures were approved by the Animal Care and Use Committee of the Provincial Government of Southern Finland and were performed in accordance with the guidelines set by the European Community Council Directive 86/609/EEC or Directive 2010/63/EU, as applicable at the time of the procedures.

Note that full information on the approval of the study protocol must also be provided in the manuscript.

Plants

Seed stocks: NA

Novel plant genotypes: NA

Authentication: NA

Magnetic resonance imaging

Experimental design

Design type: Time-dependent ex vivo diffusion MRI measurements were conducted on a mild traumatic brain injury (TBI) rat model at four weeks post-injury, designed to experimentally assess the sensitivity of the diffusion parameters to injury-induced axonal alterations.

Design specifications: dMRI measurements were performed on five adult male Sprague-Dawley rats: two sham-operated and three rats subjected to mild TBI. All animals were sacrificed four weeks post-surgery.

Behavioral performance measures: N/A

Acquisition

Imaging type(s): diffusion MRI

Field strength: 11.7 T

Sequence & imaging parameters: dMRI data were acquired using a 3d segmented PGSE sequence with the following parameters:TR = 1000 ms, TE = 51.26 ms, FA = 90 deg, data matrix 100 × 100 × 12, FOV 9 × 9 × 4 mm, in-plane resolution 90 × 90 µm2, slice thickness 333 µm with three segments.

Area of acquisition: Over major white matter tracts, specifically the cingulum (Cg) and the corpus callosum (CC)—including its splenium and body—in both ipsilateral and contralateral hemispheres of sham-operated and mild TBI rats.

Diffusion MRI ☒ Used ☐ Not used

Parameters: A total of 140 diffusion-weighted volumes were acquired, including 3 sets of 28 uniformly distributed directions at b-values of 1000, 2000, and 3000 s/mm2, with diffusion gradient parameters δ = 2.5 ms and Δ = 7, 15, 20, 30, 40 ms. Additionally, 10 volumes without diffusion weighting (b = 0) were collected.

Preprocessing

Preprocessing software: dMRI data were processed following the steps in the DESIGNER pipeline (https://github.com/NYU-DiffusionMRI/DESIGNER-v2) adapted for ex vivo dMRI rat brains. The DESIGNER denoising parameters were set to apply MP-PCA denoising with adaptive patch, applying eigenvalue shrinkage and removing partial Fourier-induced Gibbs ringing with 0.69 partial Fourier, followed by Rician bias correction. Diffusion tensor maps were computed using tools provided by the DESIGNER pipeline by fitting the diffusion kurtosis signal representation to account for non-Gaussianity effects.

Normalization: Diffusion parameters were computed after normalizing diffusion-weighted images by their corresponding non-diffusion-weighted (b = 0) images.

Normalization template: Non diffusion-weighted (b = 0) images.

Noise and artifact removal: Refer to the pre-processing step.

Volume censoring: No volume censoring.

Statistical modeling & inference

Model type and settings: Linear regression models were used to estimate the diffusion parameters by fitting axial diffusivity D(t) against 1/sqrt{t}.
Support Vector Machine classification with a linear kernel was employed to separate sham-operated and TBI animals based on the diffusion-derived or geometry-derived parameters.
No additional hyperparameter tuning was applied. All statistical inference was based on group-wise medians and nonparametric effect size.

Effect(s) tested: We tested the effect of mild TBI on axonal geometry and diffusion properties in white matter. Specifically, we assessed whether TBI leads to measurable differences in the long-time diffusivity asymptote D_infty, the amplitude c_D of its t^{-1/2} power-law decay, and geometry-derived descriptors such as axonal tortuosity and Gamma_0, which captures large-scale axon shape variability. These parameters were compared between sham-operated and TBI rats in both the corpus callosum and cingulum.

Specify type of analysis: ☐ Whole brain ☒ ROI-based ☐ Both

Anatomical location(s): We assigned sagittal slices #2–3 (of 12 total) as contralateral and slices #10–11 as ipsilateral to the injury site. Regions of interest (ROIs) for the splenium (Scc) and body (Bcc) of the corpus callosum were manually drawn using a bounding box. Within these, we included voxels with red-channel fractional anisotropy (FA) values above 0.5 for Scc and above 0.3 for Bcc. A lower threshold was applied to Bcc due to its thinner anatomy, ensuring sufficient voxel counts for statistical testing. The cingulum (Cg) ROI was drawn by tracing a consistent line of voxels just above the superior boundary of the corpus callosum in the coronal view, guided by contrast in the green FA channel.

Statistic type for inference
(See Eklund et al. 2016): We computed nonparametric effect sizes voxel-wise, using the median difference normalized by the pooled median absolute deviation. For group separability analysis between sham and TBI animals, we used voxel-level diffusion and geometric parameter values within each ROI as input features to a linear SVM.

Correction: N/A

Models & analysis

n/a	Involved in the study
☒	☐ Functional and/or effective connectivity
☒	☐ Graph analysis
☐	☒ Multivariate modeling or predictive analysis

Multivariate modeling and predictive analysis: A linear SVM was used to identify an optimal combination of diffusion or geometric parameters for classifying sham vs. TBI rats.
