# Supplementary material for: Scattering approach to diffusion quantifies axonal damage in brain injury
Source: Nat Commun. 2025 Nov 6;16:9808. doi: 10.1038/s41467-025-64793-1 (PMC12592534; doi:10.1038/s41467-025-64793-1)
Supplement: Supplementary file 4 — Source Data [file 41467_2025_64793_MOESM4_ESM.docx]

**Scattering approach to diffusion quantifies axonal damage in brain injury**

Ali Abdollahzadeh^1,2,∗^, Ricardo Coronado-Leija^1^, Hong-Hsi Lee^3^, Alejandra Sierra^2^, Els Fieremans^1^, Dmitry S. Novikov^1, ∗^

^1^Center for Biomedical Imaging, Department of Radiology, New York University School of Medicine, New York, NY, USA

^2^A.I. Virtanen Institute for Molecular Sciences, University of Eastern Finland, Kuopio, Finland

^3^Athinoula A. Martinos Center for Biomedical Imaging, Department of Radiology, Massachusetts General Hospital, Harvard, Medical School, Boston, MA, USA

^∗^ali.abdollahzadeh@uef.fi ^∗^dmitry.novikov@nyulangone.org

--------------------------------------------------------------------------------------------------------------------------------------

Due to the large file size, the source data of all figures, including the Supplementary Information, are hosted on a publicly accessible filesharing service (fairdata.fi) with an assigned DOI. The source code to generate all figures is hosted on Github with an assigned DOI.

The source data, including axonal morphology and time-dependent diffusion MRI data are publicly available at:

<https://etsin.fairdata.fi/dataset/7ab3737d-0884-400e-ab57-657e3667d52b>

The source code used to generate figures in the manuscript and Supplementary Information are publicly available at:

<https://github.com/aAbdz/scattering-to-diffusion/releases/tag/v1.0>

These repositories contain all measurements and analysis codes used to generate Figures 1–4 from the main text and Supplementary Figures S1–12.

Figures S2-S4 can be generated from codes related to Figure 3.

Figures S5-S7 can be generated from codes related to Figure 4.

All figures and their associated data/code are organized in clearly labeled directories.
